# Supplementary material for: Decades of native bee biodiversity surveys at Pinnacles National Park highlight the importance of monitoring natural areas over time
Source: PLoS One. 2019 Jan 17;14(1):e0207566. doi: 10.1371/journal.pone.0207566 (PMC6336250; doi:10.1371/journal.pone.0207566)
Supplement: S1 Table — (PDF) [file pone.0207566.s001.pdf]

Meiners et al. 2019. Decades of native bee biodiversity surveys at Pinnacles National Park highlight the importance of monitoring natural areas over time

Supporting Information

S1 Table. Names, locations, and descriptions of initial (2011-12) sampling of long-term bee monitoring study plots at Pinnacles National Park

Site/Plot Number	Site/Plot Name	GPS lat/long	Elevation	Dimensions	Habitat Type
1	McCabe Canyon Upper	36.5081, -121.156	410m	175 m x 57m	Alluvial
2	McCabe Canyon Lower	36.503, -121.156	395m	175m x 57m	Alluvial
3	Peaks View	36.4802, -121.16	290m	200m x 50m	Alluvial
4	South Wilderness	36.4683, -121.156	280m	250m x 40m	Live Oak Woodland
5	Needlegrass BOW	36.5091, -121.12	385m	200m x 50m	Blue Oak Woodland
6	Needlegrass LOW	36.509, -121.129	365m	200m x 50m	Live Oak Woodland
7	West Gate	36. 4747, -121.227	610m	175m x 57m	Blue Oak Woodland
8	Double Gates	36.4858, -121.232	535m	200m x 50m	Grassland
9	W. North Wilderness	36.4949, -121.211	430m	200m x 50m	Grassland
10	High Peaks	36.4907, -121.183	595m	175m x 50m	Blue Oak Woodland
