# Supplementary material for: Decades of native bee biodiversity surveys at Pinnacles National Park highlight the importance of monitoring natural areas over time
Source: PLoS One. 2019 Jan 17;14(1):e0207566. doi: 10.1371/journal.pone.0207566 (PMC6336250; doi:10.1371/journal.pone.0207566)
Supplement: S2 Table — Species are marked "S" for Singleton if only one specimen was collected, "R" for Rare if N≤10, and “C” for Common if N>10. Dashed vertical line marks 2002 collection as separate from original 1996–9 inventory, but still prior to recent study (2011–12). (PDF) [file pone.0207566.s002.pdf]

Meiners et al. 2019. Decades of native bee biodiversity surveys at Pinnacles National Park highlight the importance of monitoring natural areas over time

Supporting Information

S2 Table. **Full Pinnacles National Park bee species list, with relative abundance for each of seven collection years, proportion of years collected, and status as new (N) to or absent (A) from the current study.** Species are marked "S" for Singleton if only one specimen was collected, "R" for Rare if N≤10, and "C" for Common if N>10. Dashed vertical line marks 2002 collection as separate from original 1996-9 inventory, but still prior to recent study (2011-12).

Bee Taxonomy				Early Inventory				Bowls	Recent Inventory			
**Family**	**Genus**	**Subgenus**	**Species**	**1996**	**1997**	**1998**	**1999**	**2002**	**2011**	**2012**	**Prop. years present**	**New/ Absent in 2011-12**
Andrenidae	Ancylandrena		atoposoma	S							0.14	A
	Andrena	(Anchandrena)	quercina			R			R	R	0.43	
		(Belandrena)	nemophilae			R			S	R	0.43	
			palpalis		R	R					0.29	A
		(Cremnandrena)	anisochlora			C			C	R	0.43	
		(Dasyandrena)	cristata			R					0.14	A
		(Derandrena)	arctostaphylae			S		S			0.29	A
			californiensis			R		S	S	R	0.57	
			n. sp.	S	S	R			R	C	0.71	
			vandykei			C		S	S		0.43	
			viridissima						R		0.14	N
		(Diandrena)	apasta					S			0.14	A
			chalybioides							S	0.14	N
			cuneilabris							S	0.14	N
			lewisorum	S	R	S	C		C	C	0.86	
			nothocalaidis			S					0.14	A
			puthua		R	C			C	R	0.57	

	subchalybea			R	R	R	C	C	0.71	
(Erandrena)	principalis			R					0.14	A
	astragali		S	R			S	R	0.57	
	auricoma	R	R	C	R		C		0.71	
	caerulea			C			R	R	0.43	
	chlorura		R	C		R	C	C	0.71	
	dissimulans		R	C			C	C	0.57	
	misella			S					0.14	A
	nigrocaerulea	S	R	R			S	R	0.71	
	suavis			R			C	C	0.43	
	subdepressa			R			R	S	0.43	
(Genyandrena)	mackieae		R	C			R		0.43	
(Hesperandrena)	baeriae		S	S			R	R	0.57	
	compositarum						S	S	0.29	N
	escondida							R	0.14	N
(Holandrena)	cressonii ssp. infasciata		S			S			0.29	A
(Melandrena)	cerasifolii	C	C	C	S	S	C	C	1	
	aff. cerasifolii		C	C	C		C	C	0.71	
	perimelas					S			0.14	A
	sola	R	C	C	C		C	C	0.86	
(Micrandrena)	annectens			R					0.14	A
	chlorogaster		R	C		R	C	C	0.71	
	aff. ishii			C					0.14	A
	microchlora		R	C	S		R	R	0.71	
	piperi		S	R		S	R	R	0.71	
(Nemandrena)	crudeni			C		S	C	C	0.57	
	subnigripes			S					0.14	A
(Oligandrena)	macrocephala	R	C	C		C	C	C	0.86	
(Parandrena)	concinnula			S			R		0.29	
(Pelicandrena)	atypica	S	R	C	R	R	R	R	1	
(Plastandrena)	prunorum							S	0.14	N
(Psammandrena)	congrua		R	R			R	R	0.57	
(Ptilandrena)	pallidiscopa			R				R	0.29	
(Scaphandrena)	lomatii			S					0.14	A
	plana			R					0.14	A
	santaclarae		S	R			R	R	0.57	
(Scoliandrena)	cryptanthae	R					R		0.29	
	osmioides			R			R	S	0.43	
(Scrapteropsis)	biareola			R					0.14	A
(Simandrena)	angustitarsata	R	R	C	C		C	C	0.86	
	hypoleuca						S	R	0.29	N
	orthocarpi			R			R	R	0.43	
	pallidifovea	S	R	R	R		R	R	0.86	
(Thysandrena)	candida	C	C	C	C	R	C	C	1	
	knuthiana					S	C	C	0.43	
	vierecki		S	S					0.29	A

			w-scripta	R	R	C	C			S	0.71	
		(Trachandrena)	fuscicauda			S	R				0.29	A
			semipunctata		R	S			S		0.43	
		(Tylandrena)	subaustralis						S		0.14	N
			subtilis			S					0.14	A
			waldmerei			R					0.14	A
	Calliopsis	(Micronomadopsis)	fracta	R	S	R			S		0.57	
			helianthi					R	S		0.29	
			mellipes		S				R		0.29	
			trifolii	R			R				0.29	A
		(Nomadopsis)	anthidia	S	R	R		S		S	0.71	
			obscurella	R	C	R	R	C	C	R	1	
			zonalis	S	S		S				0.43	A
			smithi	C	C	C	C			S	0.71	
	Macrotera	(Macroteropsis)	arcuata			S			S	R	0.43	
	Panurginus		gracilis			C	R	C	C	C	0.71	
			melanocephalus		R	R			C	C	0.57	
			nigrellus	C	C	C		R	C	C	0.86	
			aff. occidentalis					S			0.14	A
	Perdita	(Hesperoperdita)	trisignata ssp. ornata	R	C	C	C		S	S	0.86	
		(Perdita)	claypolei ssp. limatula		C	C					0.29	A
			hirticeps ssp. hirticeps					S		R	0.29	
			n. sp. aff. holoxantha						S		0.14	N
			isocomae		S						0.14	A
			jucunda		R		S				0.29	A
			linsleyi	R	R	R	S		R		0.71	
			rhois ssp. reducta	C	C	C	S			R	0.71	
			salicis ssp. personata						R		0.14	N
		(Pygoperdita)	aureovittata ssp. stenozona		S						0.14	A
			californica			R					0.14	A
			distropica	R	C	C	C	C	C	C	1	
			micheneri ssp. micheneri			C	R	S	R		0.57	
			montereyensis	C	C	C	C	S	C	C	1	
			nitens	C	C	R			C	R	0.71	
Apidae	Anthophora	(Anthophoroides)	californica	R	C	R	R	S	R	R	1	
		(Heliophila)	columbariae			R					0.14	A
			curta	R	R	S	R		R	R	0.86	
			estebana		S						0.14	A
			flavocincta					S			0.14	A
		(Lophanthophora)	pacifica		R	C			S		0.43	
		(Melea)	bomboides	S							0.14	A
		(Mystacanthophora)	urbana	R	C	C	C	S	C	C	1	
		(Paramegilla)	centriformis	R	C	R	C		R		0.71	
		(Pyganthophora)	crotchii	R	C	R	R	S	C	C	1	
			edwardsii	R	R	R			C	C	0.71	
			platti	C	C	C	R		C	C	0.86	

Anthophorula	(Anthophorisca)	nitens		C	C			C	C	0.57	
	(Anthophorula)	albicans		C	C		R	S	C	0.71	
Apis		mellifera	R	C	C	C	C	C	C	1	
Bombus	(Crotchiibombus)	crotchii		C	C	S				0.43	A
	(Fervidobombus)	californicus	R	C	C	R	R	R	R	1	
	(Pyrobombus)	caliginosus		C				S	S	0.43	
		melanopygus	R	C	C	R	R	C	R	1	
		vandykei		R	R			R		0.43	
		vosnesenskii	R	C	C	S	S	C	R	1	
Brachynomada	(Melanomada)	melanantha							R	0.14	N
Centris	(Paracentris)	aff. californica		C	C	R				0.43	A
Ceratina	(Ceratina)	arizonensis	C	C	C	C	C	C	C	1	
	(Euceratina)	dallatorreana			S		R		C	0.43	
	(Zadontomerus)	acantha	S	C	R		R	C	C	0.86	
		hurdi	S	R	R		R	C	C	0.86	
		nanula	R	C	C	C	C	C	C	1	
		aff. nanula	S	R	S		S	R	R	0.86	
		pacifica		R	S			S	R	0.57	
		punctigena	S	C	R	C		R	S	0.86	
		sequoiae	R	C	C	C	R	C	C	1	
		tejonensis		C	R			R		0.43	
		timberlakei		R	R	R		R	S	0.71	
Diadasia		aff. ochracea		S	S		S	R	C	0.71	
		angusticeps		R	R	C	R	C	C	0.86	
		australis					S	S		0.29	
		bituberculata	R	C	R	C	C	C	C	1	
		consociata							R	0.14	N
		laticauda		C	C	R	R	C	C	0.86	
		nigrifrons						S		0.14	N
		nitidifrons		R	C	R	R	C	C	0.86	
		rinconis							R	0.14	N
Epeolus		americanus	S	R			S	R	R	0.71	
		compactus			R	S			S	0.43	
		mesillae						S	R	0.29	N
		minimus				S	S		S	0.43	
Eucera	(Synhalonia)	actuosa		S	R	R	C	C	C	0.86	
		amsinckiae	R	R	R		R	R	R	0.86	
		cordleyi		R	C		S	C	R	0.71	
		delphinii		C	R	S	S	R	R	0.86	
		dorsata		C	R	R	S	C	C	0.86	
		edwardsii	S	R	R		S		R	0.71	
		lunata			R			R	R	0.43	
		venusta ssp. carinata	C	C	C	R		R	R	0.86	
		virgata			C	S	S	C	C	0.71	
Habropoda		dammersi			R					0.14	A
		depressa	S	C	C	R	S	C	C	1	

		tristissima	R	C	R	S	R	R	C	1	
Melecta	(Melecta)	pacifica		R	R			R		0.43	
		separata			S	R	R	R	C	0.71	
	(Melectomimus)	edwardsii		R				S		0.29	
Melissodes	(Callimelissodes)	clarkiae		R				R	R	0.43	
		composita							S	0.14	N
		lupina		R	C		R	C	C	0.71	
		lustra		S						0.14	A
		n. sp. 1		R		R				0.29	A
		n. sp. 2		R	R	R	S	R	C	0.86	
		nigracauda			S					0.14	A
		plumosa		R				R	C	0.43	
		stearnsi		R	S		C	C	C	0.71	
	(Eumelissodes)	paulula		R						0.14	A
		velutina							R	0.14	N
	(Melissodes)	tepida							C	0.14	N
Neopasites	(Micropasites)	sp. 4							S	0.14	N
Nomada	(Centrias)	crotchii spp. crotchii		R	S					0.29	A
		crotchii ssp. nigrior			C			R	R	0.43	
		sp. A	S	R						0.29	A
	(Holonomada)	edwardsii spp. edwardsii		R	R				S	0.43	
	(Nomada)	sp. A	R	R	S			R	S	0.71	
		sp. AA							S	0.14	N
		sp. B	S	S	S			R	R	0.71	
		sp. BB					S	R	R	0.43	
		sp. CC					S		S	0.29	
		sp. D	C	C	C	S				0.57	A
		sp. DD						S		0.14	N
		sp. E	C	R	S	R		R	R	0.86	
		sp. EE						R		0.14	N
		sp. F	S		R				S	0.43	
		sp. FF							S	0.14	N
		sp. G						R	R	0.29	N
		sp. GG			S				R	0.29	
		sp. HH			S		S			0.29	A
		sp. I			S					0.14	A
		sp. II					R		R	0.29	
		sp. J			S			S		0.29	
		sp. Q		S	S			S		0.43	
		sp. R		R	R					0.29	A
		sp. S		S						0.14	A
		sp. T		S						0.14	A
		sp. U		C	C			R	C	0.57	
		sp. V	S	R	R		R	R	R	0.86	
		sp. W		C	C			C	C	0.57	
		sp. X			S			R	R	0.43	

			sp. Y			R		S			0.29	A
			sp. Z			C					0.14	A
	Oreopasites		aff. hurdi n.sp.		S	R	S		S	S	0.71	
			vanduzeei	R	R	S	S		S		0.71	
	Peponapis	(Peponapis)	pruinosa							R	0.14	N
	Townsendiella		ensifera		R	C	C			R	0.57	
			rufiventris			S					0.14	A
	Triepeolus		heterurus							R	0.14	N
			melanarius							S	0.14	N
			sp. P1						R	S	0.29	N
			sp. P2						R		0.14	N
			sp. P3							R	0.14	N
			sp. P4			R					0.14	A
			timberlakei		R	R	R		S	R	0.71	
			aff. timberlakei					S	R		0.29	
	Xeromelecta	(Melectomorpha)	californica	R	C	R	C		R	R	0.86	
		(Xeromelecta)	larreae	S							0.14	A
	Xylocopa	(Notoxylocopa)	tabaniformis ssp. orpifex	R	C	R	R	S	R	R	1	
Colletidae	Colletes	consors	californicus							S	0.14	N
			consors ssp. pascoensis	C	C	R	R		C	R	0.86	
		daleae	aff. algarobiae	S	C	C	R		R	C	0.86	
		simulans	fulgidus ssp. fulgidus		R	R	R			R	0.57	
			simulans ssp. nevadensis		S						0.14	A
			slevini	R	C	C	C		R	R	0.86	
	Hylaeus	(Cephalylaeus)	nunenmacheri			S	R				0.29	A
		(Hylaeus)	bisinuatus		S						0.14	A
			conspicuus				R	R		R	0.43	
			granulatus		C	C	C	S	R	R	0.86	
			mesillae ssp. cressoni	R	C	C	C	S	C	C	1	
			rudbeckiae	R					R	R	0.43	
			verticalis	R	C	C	C		R	R	0.86	
		(Paraprosopis)	aff. cookii n.sp.		R	R	R				0.43	A
			calvus	R		C	C		R	C	0.71	
			coloradensis		C	C	R				0.43	A
			n. sp.2		C	R	R		R		0.57	
			nevadensis	C	C	C	C		R	C	0.86	
			polifolii		C	C	C		R		0.57	
		(Prosopis)	aff. episcopalis	C	C	C	C		R	R	0.86	
		(Spatulariella)	punctatus				S				0.14	A
Halictidae	Agapostemon	(Agapostemon)	femoratus					R	R		0.29	
			texanus	R	R	R	C	C	C	C	1	
	Augochlorella	(Augochlorella)	pomoniella		R	R				R	0.43	
	Conanthalictus	(Phaceliapis)	bakeri		S	R	S		R	S	0.71	
			seminiger				S			S	0.29	
	Dufourea		dentipes						S		0.14	N
			leachi	S	S	C	R	R	R	C	1	

		mulleri		R	R			R		0.43	
		rhamni	R	R	R				S	0.57	
		sandhouseae		C	C			C	C	0.57	
		sparsipunctata	C	C	R	R	C	C	C	1	
		virgata	C	R	S	S		R	R	0.86	
Halictus	(Nealictus)	farinosus	C	C	C	C	C	C	C	1	
	(Odontalictus)	ligatus				R	C	C	C	0.57	
	(Protohalictus)	rubicundus					S			0.14	A
	(Seladonia)	tripartitus	C	C	C	C	C	C	C	1	
Lasioglossum	(Dialictus)	albohirtum				R		S	R	0.43	
		brunneiventre		R	R	R		C	C	0.71	
		diversopunctatum			S			R		0.29	
		hudsoniellum						S	R	0.29	N
		imbrex	R	C	C	C		C	C	0.86	
		cf. impavidum							S	0.14	N
		incompletum	R	R	C	C		C	C	0.86	
		megastictum			S			R		0.29	
		nevadense	R	C	C	C		C	C	0.86	
		perichlarum						R		0.14	N
		petrellum		S						0.14	A
		punctatoventre	R	C	C	C		C	C	0.86	
		aff. ruidosense	R	R		R				0.43	A
	(Evylaeus)	argemonis	R	C	C	C		C	C	0.86	
		giffardi					S	C	R	0.43	
		robustum		S	R	C		C	C	0.71	
	(Hemihalictus)	aspilurum		S		S		R	R	0.57	
		glabriventre	R	R	R	C		C	C	0.86	
		kincaidii	R	R	S	S		R	R	0.86	
		ovaliceps		S	R			S		0.43	
		ruficorne	R	R	C	C		C	R	0.86	
		sequoiae	S		R	C		C	C	0.71	
	(Lasioglossum)	egregium		S	R	R		C	C	0.71	
		mellipes					R	C	R	0.43	
		sisymbrii	R	C	C	C	C	C	C	1	
		titusi			S	R		C	C	0.57	
	(Sphecodogastra)	allonotum			S			R	R	0.43	
		aff. avalonense		S	R	C		C	C	0.71	
		miguelense		S		R		R	C	0.57	
		nigrescens	C	C	C	C		C	C	0.86	
		sp. 16				R		C	C	0.43	
Micralictoides		altadenae		S				R		0.29	
		ruficaudus	R	R	S			C	R	0.71	
Sphecodes		arvensiformis	S	R			S	S	R	0.71	
		sp. B	R	C	C	R		R	R	0.86	
		sp. C	R							0.14	A
		sp. D	R		S	R		R	R	0.71	

			sp. E	S	R	R	C	R	C	C	1	
			sp. F	S	R						0.29	A
			sp. I		S						0.14	A
			sp. J		R						0.14	A
			sp. K		S						0.14	A
			sp. L		S	R	C		R		0.57	
			sp. M							S	0.14	N
Megachilidae	Anthidiellum	(Loyalanthidium)	robertsoni		C	C			R		0.43	
	Anthidium	(Anthidium)	collectum	R	C	C	C	R	R	R	1	
			edwardsii							R	0.14	N
			jocosum							R	0.14	N
			maculosum		S						0.14	A
			mormonum	S	R	S	R				0.57	A
			pallidiclypeum	R	S	R	S	S			0.71	A
			utahense	C	C	C	C	C	C	C	1	
		(Callanthidium)	illustre		C	R	R		S	R	0.71	
	Ashmeadiella	(Arogochila)	aff. salviae n. sp. 2						S		0.14	N
			australis		R	R	R			S	0.57	
			salviae	R	C	C	R	S	R	C	1	
			timberlakei	C	C	C	C		R	R	0.86	
		(Ashmeadiella)	altadenae						R	R	0.29	N
			aridula					R		C	0.29	
			bucconis		C	R				R	0.43	
			cactorum ssp. basalis		R					R	0.29	
			californica ssp. californica	R	C	C	C	R	R	C	1	
			difugita ssp. emarginatula		R	R			S	R	0.57	
			femorata		R		C				0.29	A
			foveata	S	C	C	C		S		0.71	
			gillettei ssp. cismontanica		S	S					0.29	A
			meliloti		C	C					0.29	A
			pronitens		S						0.14	A
			aff. rufitarsis	R	R	R	R				0.57	A
			sonora		R	S		S		R	0.57	
			titusi		C	R	S			S	0.57	
	Atoposmia	(Atoposmia)	n. sp. 2	R	R	S			S		0.57	
			pycnognatha		C	R	S				0.43	A
		(Eremosmia)	hemizoniae						S		0.14	N
		(Hexosmia)	copelandica ssp. copelandica	R	C	R	R	S	R	R	1	
	Chelostoma	(Chelostoma)	aff. minutum n.sp.		R	R	R			S	0.57	
			californicum	C	C	C	C	S	C	R	1	
			cockerelli	C	C	C	C	R	C	C	1	
			incisulum	C	C	C	C		C	R	0.86	
			marginatum ssp. incisuloides	C	C	C	R		C	R	0.86	
			phaceliae	C	C	C	C		C	R	0.86	
			tetramerum	R	C	C	R		S	R	0.86	

Coelioxys	(Boreocoelioxys)	octodentata		R	R	S		S		0.57	
	(Coelioxys)	hirsutissima						R		0.14	N
		serricaudata	S	R	R	R	S	R	R	1	
	(Cyrtocoelioxys)	gilensis		S						0.14	A
		gonaspis		R						0.14	A
Dianthidium	(Dianthidium)	dubium ssp. dilectum		C	C	R	R	R	C	0.86	
		parvum ssp. schwarzi					R	R	R	0.43	
		pudicum ssp. consimile	S	S	S	R	R	R	C	1	
		singulare		S						0.14	A
		ulkei ssp. ulkei		R	S		S		R	0.57	
Dioxys		aurifusca		S						0.14	A
		pacifica ssp. pacifica			S		S		R	0.43	
		pomonae ssp. pomonae	R	R	R	R		C	R	0.86	
		producta ssp. cismontanica	R	R	R	R		R	S	0.86	
Heriades	(Neotrypetes)	occidentalis		C	C					0.29	A
Hoplitis	(Acrosmia)	aff. emarginata		C	R	R		S		0.57	
	(Alcidamea)	colei	R	C	R	C	R	C	C	1	
		grinnelli	R	C	R	R			R	0.71	
		producta ssp. bernardina		C				C	S	0.43	
		producta ssp. gracilis	C	C	C	C	R	C	R	1	
		sambuci	S	C	R	S			S	0.71	
	(Cyrtosmia)	hypocrita	S	C	C	R		R	R	0.86	
	(Hoplitina)	bunocephala	S		S					0.29	A
		howardi	C	C	C	C		R	R	0.86	
	(Monumetha)	albifrons ssp. maura	C	C	C	C	C	C	C	1	
		fulgida ssp. platyura	C	C	C	C	R	C	R	1	
	(Penteriades)	remotula	R	C	R		S	R	S	0.86	
	(Proteriades)	cryptanthae	R	R			S			0.43	A
		jacintana	C	C	S	R		R		0.71	
		nanula	R	C	S	R		R		0.71	
		seminigra	S	C	R	S		S		0.71	
		semirubra	R	R	R	S	R	R	R	1	
Megachile	(Argyropile)	parallela		S						0.14	A
	(Chelostomoides)	angelarum		C	C			R	R	0.57	
		davidsoni		C				S	S	0.43	
		exilis							R	0.14	N
		spinotulata		C	R					0.29	A
	(Eutricharaea)	apicalis		C	R		R	S	C	0.71	
	(Litomegachile)	coquilletti	S	C	C	S	R	C	C	1	
		gentilis		R	S				R	0.43	
		lippiae							R	0.14	N
		onobrychidis			R	S	R	R	C	0.71	
		texana	S	R	R	R				0.57	A
	(Megachile)	montivaga		R			S	S	R	0.57	
	(Megachiloides)	gravita		R		C	R	C	C	0.71	
		pascoensis	R	C	R	C	R	C	C	1	

		pseudonigra	S	R				R		0.43	
		subnigra ssp. angelica			R		R	C	R	0.57	
	(Sayapis)	fidelis		S	R				S	0.43	
		frugalis ssp. pseudofrugalis		C	C			S	C	0.57	
		inimica ssp. jacumbensis		R						0.14	A
		newberryae		R	R					0.29	A
Osmia	(Acanthosmioides)	nigrifrons		S		R				0.29	A
		nigrobarbata	R	S	S	S	S	R	S	1	
		odontogaster	R	C	C	R	S	R	C	1	
		sedula	S	C	R	R	C	R	S	1	
	(Cephalosmia)	californica	R	R	S	S		R	R	0.86	
		montana ssp. quadriceps	R	R	R	C	S	C	R	1	
	(Euthosmia)	glauca	R	C	C	C	C	C	C	1	
	(Helicosmia)	coloradensis		C	C	R		R	R	0.71	
		texana		C	S	R	R	R	R	0.86	
	(Melanosmia)	aglaia	R	C	C	C	C	C	C	1	
		atrocyanea	R	C	C	C	C	C	C	1	
		brevis	C	C	C	C	R	C	C	1	
		calla	R	C	C	C	R	C	C	1	
		cara	C	C	C	C	S	C	C	1	
		clarescens	R	C	C	R		R	R	0.86	
		cyanella	C	C	C	C	R	C	C	1	
		cyanopoda	S	C	R	R			S	0.71	
		densa	C	C	C	R	R	C	C	1	
		gabrielis	C	C	C	C	R	C	C	1	
		gaudiosa					R	C	R	0.43	
		granulosa	R	C	C	C	R	C	C	1	
		aff. hesperos						S		0.14	N
		inurbana			R	R	C	C	C	0.71	
		kincaidii	C	C	C	C	R	C	C	1	
		laeta	R	C	C	R	R	C	C	1	
		malina			S					0.14	A
		melanopleura	C	C	C	C	R	C	R	1	
		pusilla	R	C		R	R	R	R	0.86	
		aff. pusilla						S		0.14	N
		raritatis	R	R	R	R		R	R	0.86	
		regulina	R	C	C	C	C	C	C	1	
		rostrata				R				0.14	A
		sp. P1					R		R	0.29	
		tristella		R	S	R				0.43	A
		vandykei		R	R	R				0.43	A
		visenda	C	C	C	R	R	C	C	1	
	(Mystacosmia)	nemoris		R	C	C	C	C	C	0.86	
	(Osmia)	lignaria ssp. propinqua	R	C	C	S	R	C	C	1	
		ribifloris ssp. biedermannii		S	S					0.29	A
	(Pyrosmia)	nigricollis							S	0.14	N

		(Trichinosmia)	latisulcata		R	S	R		C	S	0.71	
	Protosmia	(Chelostomopsis)	rubifloris	C	C	C	C	R	C	C	1	
	Stelis	(Protostelis)	anthidioides	S		R		S	S		0.57	
			hurdi		R	R	R	R	R		0.71	
		(Stelis)	aff. foederalis n.sp.		S		R		R		0.43	
			ashmeadiellae		R	R	R		S	S	0.71	
			calliphorina		R						0.14	A
			chemsaki							R	0.14	N
			cockerelli				R		R	R	0.43	
			interrupta				S			S	0.29	
			lateralis		R	R	R		R		0.57	
			micheneri	S	R		S		R		0.57	
			montana	R	R	R	R	S	R	R	1	
			nigriventris		R	R					0.29	A
			occidentalis	S	R					S	0.43	
			subemarginata	R	R	R	R		S		0.71	
	Trachusa	(Heteranthidium)	timberlakei		R	C	C	R	R	R	0.86	
		(Trachusomimus)	perdita		C	C	C	C	C	R	0.86	
Melittidae	Hesperapis	(Amblyapis)	ilicifoliae	C	C	C	C	C	C	C	1	
		(Panurgomia)	regularis	C	C	C	C	C	C	C	1	
