# Supplementary material for: Decades of native bee biodiversity surveys at Pinnacles National Park highlight the importance of monitoring natural areas over time
Source: PLoS One. 2019 Jan 17;14(1):e0207566. doi: 10.1371/journal.pone.0207566 (PMC6336250; doi:10.1371/journal.pone.0207566)
Supplement: S3 Table — Plants are marked with “R” for rare if bee visits were fewer than 10 in that year, with “U” for uncommon if bee visits ranged between 10–100, and “C” for common when over 100 bees were collected on that plant. The last row sums the plant taxa on which bees were collected per year. Dashed vertical line marks 2002 collection as separate from original 1996–9 study, and prior to the current study. (PDF) [file pone.0207566.s003.pdf]

Meiners et al. 2019. **Decades of native bee biodiversity surveys at Pinnacles National Park highlight the importance of monitoring natural areas over time**

Supporting Information

S3 Table. **Floral taxa visited by bees at Pinnacles National Park (unique groups, identified to lowest possible level), and their relative popularity by year.** Plants are marked with "R" for rare if bee visits were fewer than 10 in that year, with "U" for uncommon if bee visits ranged between 10-100, and "C" for common when over 100 bees were collected on that plant. The last row sums the plant taxa on which bees were collected per year. Dashed vertical line marks 2002 collection as separate from original 1996-9 study, and prior to the current study.

	**Early Inventory**				**Bowls**	**Recent Inventory**	
**Plant Name**	**1996**	**1997**	**1998**	**1999**	**2002**	**2011**	**2012**
Alliaceae Allium fimbriatum						R	
Alliaceae Allium lacunosum		U	U	R			
Alliaceae Allium lacunosum var.micranthum							R
Alliaceae Allium sp.	U	U	R				
Anacardiaceae Toxicodendron diversilobum						R	
Apiaceae Anthriscus caucalis						R	
Apiaceae Apiaceae sp.		R	U				
Apiaceae Apiaceae sp. (yellow)			R			R	
Apiaceae Lomatium dasycarpum							R
Apiaceae Lomatium sp.		U	R				R
Apiaceae Lomatium utriculatum			R			U	R
Apiaceae Perideridia californica			R				
Apiaceae Sanicula crassicaulis						R	
Apiaceae Sanicula sp.		R					
Apiaceae Sanicula tuberosa						R	R
Asclepiadaceae Asclepias sp.			U				
Asteraceae Achillea millefolium		R				R	R
Asteraceae Agoseris grandiflora						R	
Asteraceae Agoseris sp.		R					

Asteraceae Anaphalis margaritacea		R					
Asteraceae Asteraceae sp.		R	U				
Asteraceae Asteraceae sp. (yellow)			R				
Asteraceae Baccharis pilularis		U	U				
Asteraceae Baccharis salicifolia	U	R		U		U	U
Asteraceae Carduus tenuiflorus		U					
Asteraceae Centaurea melitensis						R	R
Asteraceae Centaurea solstitialis		U	U		R		R
Asteraceae Chaenactis glabriuscula		R				C	U
Asteraceae Cirsium occidentale		R	U	R		R	R
Asteraceae Cirsium sp.		U	R				
Asteraceae Cirsium vulgare		R	R				
Asteraceae Erigeron foliosus		U					
Asteraceae Erigeron foliosus var.foliosus							R
Asteraceae Erigeron petrophilus		U					
Asteraceae Eriophyllum confertiflorum		U	U	U		R	R
Asteraceae Eriophyllum lanatum			R				
Asteraceae Eriophyllum multicaule							R
Asteraceae Eriophyllum sp.		U					
Asteraceae Euthamia occidentalis		R					
Asteraceae Gnaphalium bicolor						R	
Asteraceae Gnaphalium californicum						R	R
Asteraceae Hemizonia lobbii		U					
Asteraceae Heterotheca sessiliflora		U					
Asteraceae Hypochaeris glabra						R	
Asteraceae Hypochaeris radicata						U	R
Asteraceae Lasthenia californica	R	U	C			U	U
Asteraceae Layia hieracioides		R					
Asteraceae Lessingia tenuis				U			
Asteraceae Madia sp.		R					
Asteraceae Malacothrix californica						R	
Asteraceae Microseris douglasii						R	
Asteraceae Packera breweri						R	
Asteraceae Pectis papposa		R					
Asteraceae Senecio flaccidus		R					
Asteraceae Senecio sp.		U					
Asteraceae Stephanomeria virgata ssp.pleurocarpa			R				
Asteraceae Wyethia helenioides	R					U	U
Asteraceae Wyethia sp.				R			
Boraginaceae Amsinckia menziesii	U			R	R	U	U
Boraginaceae Amsinckia menziesii var.menziesii		U	U	R		R	
Boraginaceae Amsinckia sp.					U		
Boraginaceae Cryptantha sp.	U	C	U	R			R
Boraginaceae Emmenanthe penduliflora			R	U			
Boraginaceae Eriodictyon sp.			R				
Boraginaceae Eriodictyon tomentosum	U	C	U	C		U	U
Boraginaceae Heliotropium curassavicum		U				U	U
Boraginaceae Nemophila menziesii var.integrifolia		U	U				
Boraginaceae Nemophila menziesii var.menziesii						R	R
Boraginaceae Phacelia brachyloba		U					
Boraginaceae Phacelia californica		U					
Boraginaceae Phacelia distans		U	U				R
Boraginaceae Phacelia imbricata		U	U	U		R	
Boraginaceae Phacelia malvifolia			R	U			

Boraginaceae Phacelia ramosissima		U	U	R			
Boraginaceae Phacelia ramosissima var.ramosissima						R	
Boraginaceae Phacelia sp.	U	C	U	U			R
Boraginaceae Phacelia sp. (white)	U						
Boraginaceae Pholistoma auritum	U	C	C	R			R
Boraginaceae Pholistoma auritum var.auritum						U	R
Boraginaceae Pholistoma membranaceum			U			R	R
Boraginaceae Plagiobothrys canescens						U	
Boraginaceae Plagiobothrys nothofulvus			C				
Boraginaceae Plagiobothrys sp.		R	U		U		R
Brassicaceae Brassica nigra		C	U	U			
Brassicaceae Brassicaceae sp.					R		
Brassicaceae Cardamine californica			U				
Brassicaceae Cardamine californica var.californica			U			R	
Brassicaceae Erysimum capitatum var.capitatum						R	
Brassicaceae Erysimum sp.		R					
Brassicaceae Hirschfeldia incana						C	C
Brassicaceae Rorippa nasturtium-aquaticum		U		R			
Brassicaceae Thysanocarpus curvipes			C				
Brassicaceae Thysanocarpus laciniatus							R
Caprifoliaceae Lonicera hispidula			U				
Caprifoliaceae Lonicera sp.			U				R
Caprifoliaceae Lonicera subspicata var.denudata						R	
Caprifoliaceae Sambucus mexicana						R	U
Chenopodiaceae Chenopodium californicum						R	
Convolvulaceae Calystegia collina						R	U
Convolvulaceae Calystegia collina ssp.venusta						R	
Convolvulaceae Calystegia purpurata							R
Convolvulaceae Calystegia sp.			R				
Convolvulaceae Calystegia subacaulis	R	U					
Convolvulaceae Convolvulus arvensis						R	
Crassulaceae Dudleya cymosa		R	R	R			
Crassulaceae Dudleya sp.		R					
Crassulaceae Sedum spathulifolium		R					
Cuscutaceae Cuscuta californica		R					
Ericaceae Arctostaphylos pungens			C			U	
Ericaceae Arctostaphylos sp.			R		U		
Euphorbiaceae Euphorbia sp.			R				
Fabaceae Glycyrrhiza lepidota		R					
Fabaceae Lotus humistratus/wragelianus						R	
Fabaceae Lotus micranthus						R	
Fabaceae Lotus purshianus		U				R	
Fabaceae Lotus scoparius	U	C	C				
Fabaceae Lotus scoparius var.scoparius						C	U
Fabaceae Lotus sp.		U	U	R			
Fabaceae Lotus wrangelianus			U				
Fabaceae Lupinus albifrons	R	U	U			R	U
Fabaceae Lupinus albifrons var.albifrons						U	U
Fabaceae Lupinus bicolor		R					
Fabaceae Lupinus concinnus						R	
Fabaceae Lupinus microcarpus var.densiflorus						R	
Fabaceae Lupinus sp.	R	R	R				
Fabaceae Melilotus indicus		R				U	R
Fabaceae Trifolium albopurpureum						R	

Fabaceae Trifolium depauperatum						R	
Fabaceae Trifolium gracilentum var.gracilentum						R	
Fabaceae Trifolium microcephalum		R					R
Fabaceae Trifolium sp.	U	U	R				
Fabaceae Trifolium willdenovii			U			R	R
Fabaceae Vicia sp.			R				
Fabaceae Vicia villosa		U	R			U	U
Fagaceae Quercus agrifolia						R	R
Fagaceae Quercus agrifolia var.agrifolia						U	U
Fagaceae Quercus douglasii						R	R
Fagaceae Quercus lobata						R	
Fagaceae Quercus sp.	U	R	R			R	R
Fumariaceae Dicentra chrysantha		U	R	U			R
Fumariaceae Dicentra sp.			U				
Geraniaceae Erodium botrys						R	R
Geraniaceae Erodium brachycarpum						U	U
Geraniaceae Erodium cicutarium			R			U	U
Geraniaceae Erodium sp.		R					
Hippocastanaceae Aesculus californica		R	R			R	
Lamiaceae Lamium amplexicaule							R
Lamiaceae Lepechinia calycina		U	U	R		R	U
Lamiaceae Marrubium vulgare						U	U
Lamiaceae Mentha spicata		U					
Lamiaceae Mentha suaveolens		U					
Lamiaceae Monardella lanceolata		R					
Lamiaceae Monardella sp.			R				
Lamiaceae Monardella villosa		R				R	R
Lamiaceae Salvia columbariae						R	
Lamiaceae Salvia mellifera	R	U	U	U		U	R
Lamiaceae Stachys bullata		U	R	U		R	U
Lamiaceae Trichostema lanatum	R	U	U	R		R	R
Lamiaceae Trichostema lanceolatum	R	U					R
Liliaceae Bloomeria crocea		U	U			U	
Liliaceae Brodiaea sp.		R					
Liliaceae Brodiaea terrestris		R	U			R	R
Liliaceae Calochortus venustus		U	U	U		U	U
Liliaceae Dichelostemma capitatum		U				R	U
Liliaceae Triteleia hyacinthina		R					
Liliaceae Triteleia lugens		R	U				U
Liliaceae Zigadenus fremontii		R	R				
Liliaceae Zigadenus venenosus							R
Malvaceae Eremalche parryi				U			
Malvaceae Malacothamnus aboriginum		U	U			U	U
Oleaceae Fraxinus dipetala		R				R	
Onagraceae Camissonia sp.	R	U	R	R			R
Onagraceae Clarkia affinis		R				R	
Onagraceae Clarkia cylindrica		R	R				R
Onagraceae Clarkia modesta		R	R	R		R	
Onagraceae Clarkia purpurea	R	U	U	U		C	U
Onagraceae Clarkia similis		R					
Onagraceae Clarkia sp.	U	U	U	U			
Onagraceae Clarkia speciosa						R	R
Onagraceae Clarkia unguiculata		C	C	U		C	C
Onagraceae Epilobium canum			R				

Orobanchaceae Castilleja affinis			R			R	
Orobanchaceae Castilleja exserta		R	R			R	
Orobanchaceae Castilleja sp.		R	R				
Orobanchaceae Pedicularis densiflora						R	
Orobanchaceae Pedicularis sp.			R				
Orobanchaceae Triphysaria pusilla						R	
Papaveraceae Dendromecon rigida		U	R	R		R	
Papaveraceae Eschscholzia californica	U	C	U	C	R	C	C
Papaveraceae Eschscholzia sp.					R		
Papaveraceae Meconella linearis		R					
Papaveraceae Platystemon sp.			R				
Phyrmaceae Mimulus aurantiacus	R	C	R	C		U	R
Phyrmaceae Mimulus guttatus				R		U	R
Phyrmaceae Mimulus pilosus		U					
Phyrmaceae Mimulus sp.		R	R				
Pinaceae Pinus sabiniana						R	R
Plantaginaceae Antirrhinum multiflorum		U	R				
Plantaginaceae Antirrhinum sp.			R	R			
Plantaginaceae Collinsia heterophylla	U	C	C	U		R	U
Plantaginaceae Collinsia parviflora		U					
Plantaginaceae Keckiella breviflora		U	U				
Plantaginaceae Penstemon centranthifolius		U	R			U	U
Plantaginaceae Penstemon heterophyllus		R	U			U	U
Plantaginaceae Plantago erecta						R	
Plantaginaceae Veronica anagallis-aquatica		U				R	
Polemoniaceae Gilia achilleifolia			U				
Polemoniaceae Gilia angelensis		U					
Polemoniaceae Gilia capitata	R	R				R	
Polemoniaceae Gilia sp.		R	U				
Polemoniaceae Linanthus parviflorus						R	
Polemoniaceae Linanthus sp.		R	R				R
Polemoniaceae Navarretia hamata							U
Polemoniaceae Navarretia sp.			R				
Polygonaceae Chorizanthe douglasii	R	U		C		U	U
Polygonaceae Eriogonum elongatum		U					
Polygonaceae Eriogonum fasciculatum	U	C	C	U		R	
Polygonaceae Eriogonum fasciculatum var.foliolosum						C	C
Polygonaceae Eriogonum gracile		R					
Polygonaceae Eriogonum nortonii		R				R	
Polygonaceae Eriogonum sp.			U				
Polygonaceae Eriogonum vimineum		U					
Polygonaceae Polygonum punctatum		R					
Polygonaceae Polygonum sp.				U			
Portulacaceae Claytonia perfoliata		R	U			U	R
Portulacaceae Montia fontana		R					
Primulaceae Anagallis arvensis			R				R
Primulaceae Dodecatheon clevelandii							R
Primulaceae Dodecatheon clevelandii ssp.patulum						R	
Primulaceae Dodecatheon sp.		R					
Ranunculaceae Clematis lasiantha		R				R	R
Ranunculaceae Clematis sp.		R					
Ranunculaceae Delphinium hesperium							R
Ranunculaceae Delphinium hesperium ssp.pallescens						R	
Ranunculaceae Delphinium parryi						R	R

Ranunculaceae Delphinium parryi/patens						R	
Ranunculaceae Delphinium sp.		U	R	R			
Ranunculaceae Ranunculus californicus		R	U			C	U
Rhamnaceae Ceanothus cuneatus		R	C	U		U	
Rhamnaceae Ceanothus cuneatus var.cuneatus						U	U
Rhamnaceae Ceanothus sp.			R		R		
Rhamnaceae Rhamnus ilicifolia		R	U	C		R	R
Rhamnaceae Rhamnus sp.						R	
Rosaceae Adenostoma fasciculatum	U	U	C	R		C	C
Rosaceae Cercocarpus betuloides		R				R	R
Rosaceae Drymocallis glandulosa							R
Rosaceae Heteromeles arbutifolia		U				R	
Rosaceae Prunus ilicifolia		U	R	C		R	
Rosaceae Rosa californica		R				U	R
Rosaceae Rubus parviflorus				R			
Rosaceae Rubus sp.		R					
Rosaceae Rubus ursinus		U					
Rubiaceae Galium sp.		R					
Salicaceae Salix exigua						U	U
Salicaceae Salix laevigata						U	R
Salicaceae Salix lasiolepis						U	U
Salicaceae Salix sp.		U	C				
Saxifragaceae Lithophragma affine			R				
Saxifragaceae Saxifraga californica			U				
Scrophulariaceae Scrophularia californica		R		R			
Solanaceae Solanaceae sp.					R		
Solanaceae Solanum umbelliferum		U	R			U	U
Valerianaceae Plectritis ciliosa							R
Valerianaceae Plectritis macrocera			U			U	
Valerianaceae Plectritis sp.		R	U				
Verbenaceae Verbena lasiostachys var.scabrida						R	R
Verbenaceae Verbena sp.							R
Violaceae Viola pedunculata		R	U			U	U
**Count of unique floral taxa on which bees were collected in each year of Pinnacles study (sampling effort not equal):**	**30**	**142**	**115**	**49**	**11**	**128**	**102**
