# Supplementary figures and images for: Decades of native bee biodiversity surveys at Pinnacles National Park highlight the importance of monitoring natural areas over time
Source: PLoS One. 2019 Jan 17;14(1):e0207566. doi: 10.1371/journal.pone.0207566 (PMC6336250; doi:10.1371/journal.pone.0207566)

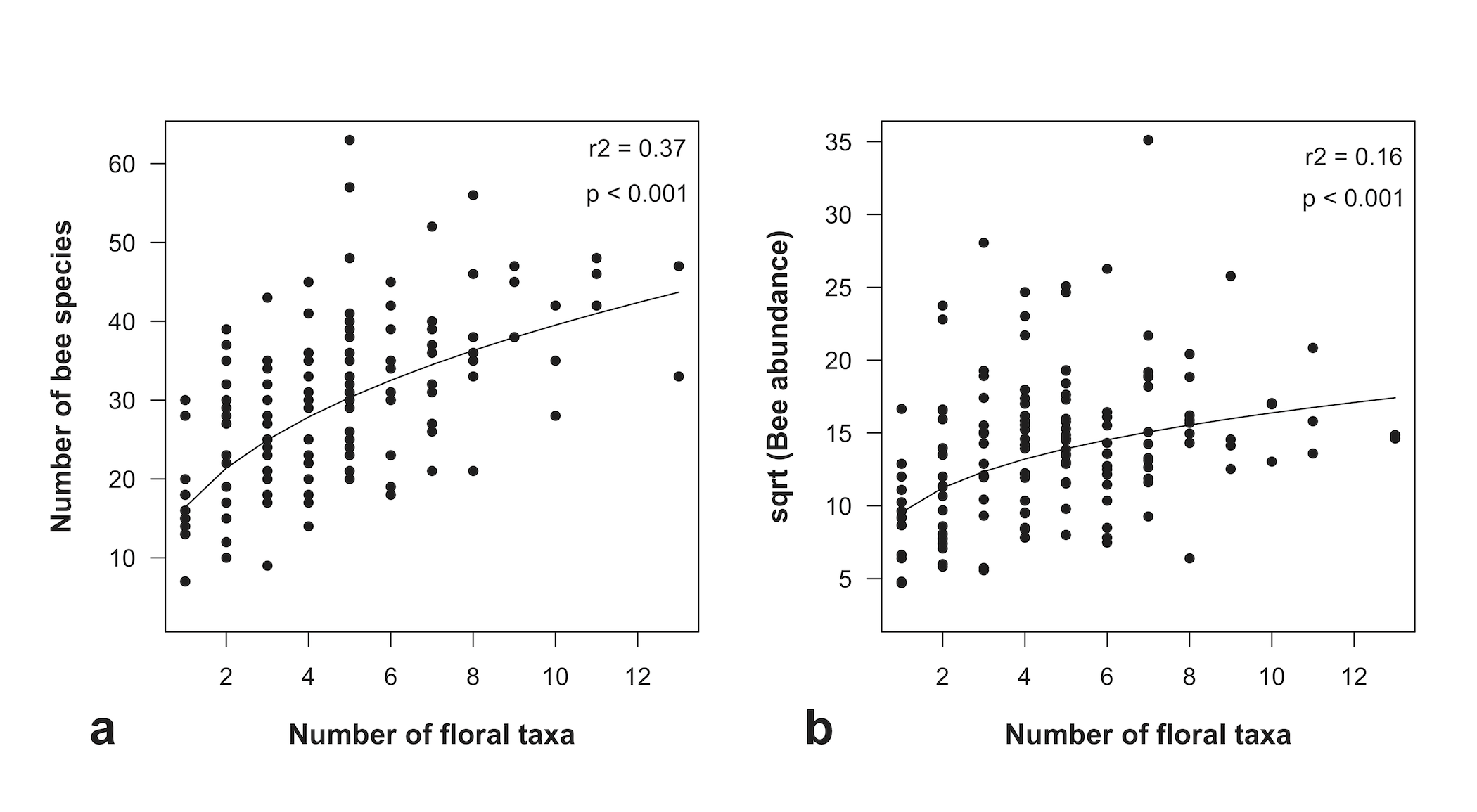

Supplement: S1 Fig — Shown with power-law model (black line; (a) BR = exp(2.79 + 0.38*log(FR)); R2 = 0.37, p<0.01; (b) BA = exp(2.26 + 0.23*log(FR)); R2 = 0.16, p<0.01). (TIFF) [file pone.0207566.s004.tiff]
