# Supplementary material for: Decades of native bee biodiversity surveys at Pinnacles National Park highlight the importance of monitoring natural areas over time
Source: PLoS One. 2019 Jan 17;14(1):e0207566. doi: 10.1371/journal.pone.0207566 (PMC6336250; doi:10.1371/journal.pone.0207566)
Supplement: S1 File — All data contributing to analyses presented in this manuscript that are not already included within tables above are shared in a zip file, along with a metadata document that details the meaning of each column, row, and variable name and which figure or table that data was used to create. This information, along with code used for analyses, is also shared on Github at https://github.com/beecycles/pinnacles_bee_biodiversity. (ZIP) [file pone.0207566.s005.zip › data_metadata/Meiners_PINNBiodiversity_metadata.docx]

**Three decades of native bee biodiversity surveys at Pinnacles National Park highlight the importance of monitoring natural areas over time**

Meiners, Joan M.^1*^, Griswold, Terry L.^2^, Carril, Olivia Messinger ^3^

### Metadata for included data files

**File name: loc_days_species.csv**

Used to make map for Fig 1

dimensions: 122 rows, 8 columns

Columns:

**Plot**: categorical; descriptive; one of four habitat types (Alluvial, BlueOak, LiveOak, or Grassland) that applies to ten plots surveyed

**Category**: categorical: whether location on map was a Plot; Recent (locations outside of a plot included in recent survey); Bowls (three locations of 2002 survey that used bowl traps only); or Early (locations of survey that took place in the 1990s)

**NewLands**: categorical; indicates whether the location was new to the park-owned lands as of the recent survey

**Sample Location**: name of plot or point location surveyed

**Latitude**

**Longitude**

**Days Sampled**: numeric; number of different days

**Bee Species Richness**: numeric; number of different bee species recorded at that location

**File name: Species_list.csv**

Used for Fig 2a and Tables 1-2

dimensions: 455 rows, 14 columns

Columns:

**Family**: categorical; taxonomic family determination

**Genus**: categorical; taxonomic genus determination

**Subgenus**: categorical; taxonomic subgenus determination

**Species**: categorical; taxonomic species determination

**1996**: categorical; abundance of bees in the year 1996; S = singleton, only one specimen, R = rare, less than 10, C = common, 10 or more

**1997**: categorical; abundance of bees in the year 1997; S = singleton, only one specimen, R = rare, less than 10, C = common, 10 or more

**1998**: categorical; abundance of bees in the year 1998; S = singleton, only one specimen, R = rare, less than 10, C = common, 10 or more

**1999**: categorical; abundance of bees in the year 1999; S = singleton, only one specimen, R = rare, less than 10, C = common, 10 or more

**2002**: categorical; abundance of bees in the year 2002; S = singleton, only one specimen, R = rare, less than 10, C = common, 10 or more

**2011**: categorical; abundance of bees in the year 2011; S = singleton, only one specimen, R = rare, less than 10, C = common, 10 or more

**2012**: categorical; abundance of bees in the year 2012; S = singleton, only one specimen, R = rare, less than 10, C = common, 10 or more

**Prop. years present**: numeric; number of years at least one specimen in that species was collected, divided by seven total years

**New/ Absent in 2011-12**: categorical; A = absent in 2011-12, N = new in 2011-12

**Num_years**: total number of years, out of seven, that species was collected

**File name: SpeciesinFamily_450.csv**

Used for Fig 2b

dimensions: 6 rows, 13 columns

Columns:

**Family**: categorical; taxonomic family determination

**All96**: numeric; number of species in that family recorded in that year (1996)

**All97**: numeric; number of species in that family recorded in that year (1997)

**All98**: numeric; number of species in that family recorded in that year (1998)

**All99**: numeric; number of species in that family recorded in that year (1999)

**All02**: numeric; number of species in that family recorded in that year (2002)

**All11**: numeric; number of species in that family recorded in that year (2011)

**All12**: numeric; number of species in that family recorded in that year (2012)

**New97**: numeric; number of species in that family newly to the park species list as of that year (1997)

**New98**: numeric; number of species in that family newly to the park species list as of that year (1998)

**New99**: numeric; number of species in that family newly to the park species list as of that year (1999)

**New02**: numeric; number of species in that family newly to the park species list as of that year (2002)

**New11**: numeric; number of species in that family newly to the park species list as of that year (2011)

**New12**: numeric; number of species in that family newly to the park species list as of that year (2012)

**File name: samples.csv**

Used for Fig 2b

dimensions: 149 rows, 334 columns

Crosstab file

**Rows** = plot sample ID

**Columns** = bee species ID

**Cell value** = numeric; abundance values

**File name: floral_richness.csv**

Used for S1 Fig

dimensions: 149 rows, 4 columns

Columns:

**floralRichness**: numeric; number of different floral taxa recorded in given plot sample

**beeRichness**: numeric; number of different bee species recorded in given plot sample

**beeAbundance**: numeric; number of different bee specimens collected in given plot sample

**sqrtAbun**: numeric; square root of beeAbundance column

**All other files used for analyses are included as tables in the supporting information document**
